# Supplementary material for: Identification and functional analysis of non-coding regulatory small RNA FenSr3 in Bacillus amyloliquefaciens LPB-18
Source: PeerJ. 2023 May 15;11:e15236. doi: 10.7717/peerj.15236 (PMC10194069; doi:10.7717/peerj.15236)
Supplement: Supplemental Information 4 [file peerj-11-15236-s004.zip › KO/CK-vs-T1_map/map04071.html]

KEGG PATHWAY: Sphingolipid signaling pathway - Reference pathway


|  |  |
| --- | --- |
| **Sphingolipid signaling pathway - Reference pathway** |  |

[
Pathway menu
| Organism menu
| Pathway entry
| Show description
| User data mapping
]

Sphingomyelin (SM) and its metabolic products are now known to have second messenger functions in a variety of cellular signaling pathways. Particularly, the sphingolipid metabolites, ceramide (Cer) and sphingosine-1-phosphate (S1P), have emerged as a new class of potent bioactive molecules. Ceramide can be generated de novo or by hydrolysis of membrane sphingomyelin by sphingomyelinase (SMase). Ceramide is subsequently metabolized by ceramidase to generate sphingosine (Sph) which in turn produces S1P through phosphorylation by sphingosine kinases 1 and 2 (SphK1, 2). Both ceramide and S1P regulate cellular responses to stress, with generally opposing effects. S1P functions as a growth and survival factor, acting as a ligand for a family of G protein-coupled receptors, whereas ceramide activates intrinsic and extrinsic apoptotic pathways through receptor-independent mechanisms.

|  |  |  |
| --- | --- | --- |
| Reference pathway | 184% 150% 122% 100% 82% 67% 55% | 图片下载 |
